# A case report of phacolytic glaucoma in lens cortex behind posterior capsule: A CARE-compliant article

**DOI:** 10.1097/MD.0000000000035784

**Published:** 2023-11-03

**Authors:** Rui-Juan Han, Ai-Jun Tian, Yi-Xiang Wu, Mei-Mei Zhou, Ying-Wei Guo, Ming-Yu Ren

**Affiliations:** a Department of Glaucoma, Hebei Eye Hospital, Hebei Provincial Key Laboratory of Ophthalmology, Hebei Provincial Clinical Research Center for Eye Diseases, Xingtai, Hebei Province, China.

**Keywords:** case report, lens cortex, phacolytic glaucoma, posterior capsule, spontaneous capsule rupture

## Abstract

**Rationale:**

Phacolytic glaucoma (PLG), a secondary open-angle glaucoma caused by high molecular weight proteins leaking through the capsule of a hypermature cataract. Leakage of liquefied lens cortex behind the posterior capsule is rare. In this paper, we review a case of phacolytic glaucoma in the lens cortex behind posterior capsule.

**Patient concerns:**

This case report describes a 79-year-old male patient with a 7-year history of progressive blurred vision and a 1-day history of distended in his left eye. He underwent phacoemulsification combined with intraocular lens implantation at our facility 7 years ago.

**Diagnoses:**

The patient had lower vision (light perception vision) and increased intraocular pressure (IOP) (60 mmHg) in the left eye. Auxiliary inspection found that the left eye had deep anterior chamber depth (around 1 corneal thickness of the peripheral AC angle) as well as vitreous and aqueous humor opacity in the left eye. Combining the clinical symptoms and examinations, we made the diagnosis of PLG in the left eye.

**Interventions:**

The patient underwent trabeculectomy and extracapsular cataract extraction of the left after a stable ocular condition, during the operation to see that white chyous cortex was visible under the posterior capsule and posterior capsule membrane of the lens was avulsed circularly.

**Outcomes:**

The postoperative condition was stable. During the follow up of 3 months, the IOP of the left eye was stable without ocular discomfort.

**Lessons:**

This case reported a patient with phacolytic glaucoma in the lens cortex behind posterior capsule who underwent successful surgery, indicating spontaneous capsule rupture can occur in the posterior capsules in PLG and when this situation is detected during the operation, the posterior capsule tearing method can be applied to absorb the lens cortex sticking at the posterior surface of the posterior capsule.

## 1. Introduction

Lens-induced glaucoma (LIG) is a type of secondary glaucoma in which the lens plays an important pathogenic role, either as a result of increased thickness, change in position, or an inflammatory process.^[1]^ Among them is phacolytic glaucoma (PLG), a secondary open-angle glaucoma caused by high molecular weight proteins leaking through the capsule of a hypermature cataract.^[2]^ Normally, the lens pocket often serves as a barrier to prevent leakage of lens protein. However, as people age and develop cataracts, the composition of the lens changes due to an increase in macromolecular lens protein and the appearance of small cracks in the lens capsule. This causes high molecular weight protein to leak into the anterior chamber (AC), which then blocks the anterior chamber angle.^[3]^ Crystallins also stimulate inflammatory and macrophage responses, where they are phagocytized by macrophages, further blocking the anterior chamber angle, and increasing the intraocular pressure (IOP).^[4,5]^

With the advancement of medical technology and medical conditions, PLG has become increasingly rare in recent years.^[6]^ While a large number of liquefied lens cortex-filled the anterior chamber is more common in PLG,^[3,7,8]^ leakage of liquefied lens cortex behind the posterior capsule is rare.^[9,10]^ A recent case of PLG was admitted to our department, and the report is as follows.

## 2. Case report

This case report complied with the Declaration of Helsinki and was approved by the Medical Ethic Committee of Hebei Eye Hospital. Written informed consent was obtained from the patient and his family members. This case report was in accord with CARE guidelines.

**Figure 1.**
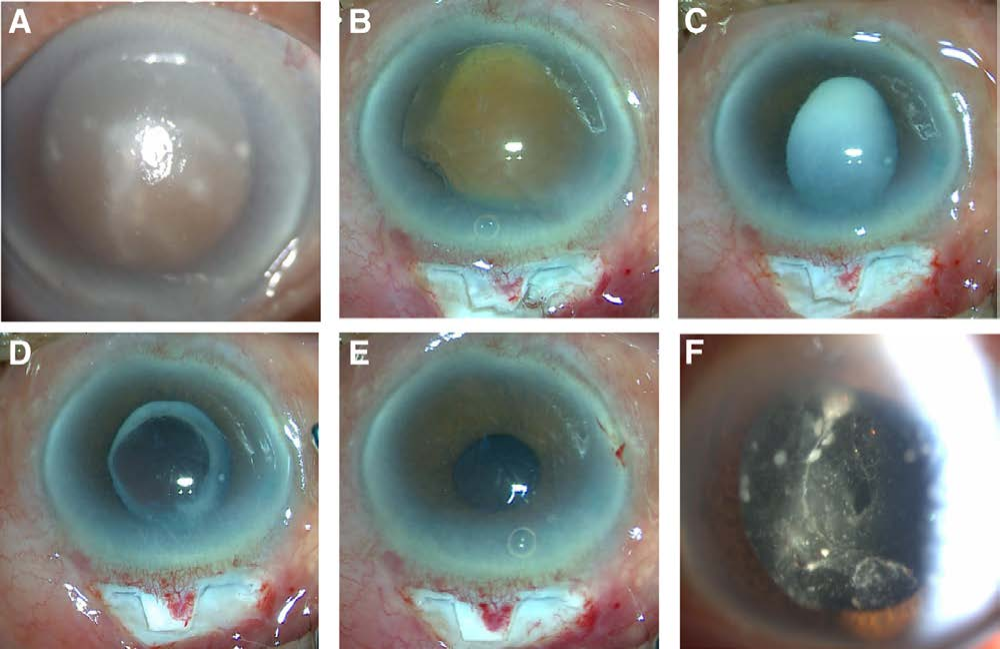
(A) Diffuse corneal edema, Anterior lens capsule dispersed in white calcified flakes, cortex releasing, and brown nuclei deposited below and in a sun-setting shape after mydriasis, in a 79-year-old east Asian male patient. (B) After intraoperative continuous curvilinear capsulorhexis, water separation and snare were used to completely take outremove the lens nucleus completely. (C) White chyous cortex was visible under the posterior capsule. (D) After intraoperative continuous curvilinear capsulorhexis, water separation and snare were used to completely take out the lens nucleus completely. (E) No intraocular lens (IOL) was implanted owing to the poor stability of the lens capsule. (F) The anterior hyaloid membrane had a slit pore with a diameter of about 1 mm, but no vitreous incarceration was observed.

A 79-year-old male patient was admitted to our hospital with a 7-year history of progressive blurred vision and a 1-day history of distended in his left eye. Seven years before, the patient underwent phacoemulsification combined with intraocular lens implantation at our facility. The patient had a vision of 0.25 in the right eye and light perception vision in the left eye. On the noncontact tonometer, the IOP was 19 mm Hg in the right eye and 60 mm Hg in the left eye. The left eye had moderate conjunctival congestion, diffuse corneal edema, and deep anterior chamber depth (around 1 corneal thickness of the peripheral AC angle) with white and colored granular substances floating. The pupil was 2 mm in diameter and unresponsive to light, with the anterior lens capsule dispersed in white calcified flakes, cortex releasing, and brown nuclei deposited below and in a sun-setting shape after mydriasis (Fig. 1A). A/B-scan revealed vitreous opacity in the left eye. Ultrasound biomicroscopy revealed an AC depth of 4.02 mm in the right eye and 3.33 mm in the left eye. Meanwhile, the left eye had aqueous humor opacity. The patient was diagnosed with PLG in the left eye.

The patient was administered antiglaucoma medications for IOP control. The anterior chamber angle was opened via gonioscopy. After a stable ocular condition, we performed trabeculectomy and extracapsular cataract extraction on the left. After intraoperative continuous curvilinear capsulorhexis, water separation and snare were used to completely take out the lens nucleus completely (Fig. 1B). White chyous cortex was visible under the posterior capsule (Fig. 1C). The Posterior capsule membrane of the lens was avulsed circularly (Fig. 1D). Remove the lens cortex which sticks to the posterior surface of the posterior capsule. A small amount of cortex remained closely attached to the posterior surface of the posterior capsule and was not sucked out. No intraocular lens was implanted owing to the poor stability of the lens capsule (Fig. 1E). Deep scleral tissue, including the trabeculae and schlemm canal, was removed. Postoperative day 1: The patient had a vision of FC/20 cm in the left eye. The IOP was 11 mm Hg. The conjunctival incision was well matched, the suture was in place, the filter bubble was dispersed, and a few folds existed in the posterior elastic layer of the central cornea. The anterior chamber was deep with aqueous flarea (++). The pupil was 1 mm in diameter and unresponsive to light. The lens was absent. After mydriasis, the capsule of the lens was complete, with a small amount of calcified cortical residue in the anterior and posterior capsule of the lens; besides, the posterior lens capsule was avulsed rounded with a diameter of about 3 mm. The anterior hyaloid membrane had a slit pore with a diameter of about 1 mm, but no vitreous incarceration was observed (Fig. 1F). The clear boundary and normal color of the optic disc loomed. The postoperative condition was stable, and the IOP fluctuated between 11 and 17 mm Hg. Optometry results showed + 15.00 D = 0.1 in the left eye. Since the patient was older and the visual acuity of the contralateral eye was satisfactory, we did not consider a second-stage intraocular lens suspension.

The patient was followed up for 3 months. The IOP of the left eye was stable, fluctuating between 13- and 18-mm Hg, without ocular discomfort.

## 3. Discussion

PLG was first described by von Reuss in 1900 as an acute increase in intraocular pressure caused by an open-angle and mature or overmature cataract.^[11]^ In 1955, Flock et al named it PLG. PLG presents acutely with conjunctival hyperemia, corneal edema, aqueous flarea, small particles commonly circulating in aqueous humor, often with hypopyon, polychromatic hyperrefringent or crystalline particle in the anterior chamber, and hypermature cataract, behind a semi, dilated pupil with open-angle.^[12,13]^

Typical cases of PLG can be easily diagnosed. Spontaneous rupture of the anterior lens capsule is more common, whereas spontaneous rupture of the posterior lens capsule is uncommon. In the present case, we observed white and colored granular substances floating in the AC, white calcified spots in the anterior capsule of the lens, chylous cortex, and sun-setting nucleus. During the operation, most of the lens cortex was found stuck to the posterior surface of the posterior capsule. This was thought to be the result of lens dislocation, causing the liquefied lens cortex to penetrate the AC from the spontaneously ruptured anterior capsule, subsequently entering the posterior capsule via the ruptured suspensory ligament. However, no clear signs of suspensory ligament rupture were found. Therefore, we excluded the possibility of lens subluxation. There was also a possibility of spontaneous rupture of the posterior capsule and the liquefied lens cortex leaking into the posterior surface of the posterior capsule. In 2019, Santos Diaz et al suggested that this increase in permeability could also be observed in the posterior capsule.^[10]^ As a result, PLG was diagnosed.

While the primary management of PLG entails urgently controlling IOP, the definitive treatment involves removing the primary cause – the crystalline lens – with or without intraocular lens implantation.^[14]^ For our case, we used the operation plan of extracapsular cataract extraction combined with trabeculectomy considering the advanced age of the patient, remote home address, limited economic conditions, and poor compliance with follow-up. This means that for other cases, it is necessary to comprehensively evaluate the patients and propose customized treatment options. In the present case, only a small amount of liquefied cortex was observed in the capsule, and the lens nucleus was completely delivered after intraoperative circular capsulorhexis. The lens cortex was subsequently found stuck to the posterior surface of the posterior capsule, posterior curvilinear capsulorhexis, and removed the lens cortex which sticking to the posterior surface of the posterior capsule. Elsewhere, Mansoori reported vitreous opacities in 1 patient with phacolytic glaucoma, which was observed intraoperatively and resolved within 12 weeks after cataract surgery, without any surgical intervention.^[9]^ Hernández-Guzmán described a liquefied lens cortex leak to the posterior surface of the posterior capsule in 1 patient with phacolytic glaucoma, which was discovered during a posterior capsulotomy using a vitrector.^[10]^ In our case, intraoperative lens cortex leakage to the posterior surface of the posterior capsule was found. The posterior capsule was treated by continuous circular capsulorhexis, and removed from the lens cortex slowly. The lens cortex, which closely sticks to the posterior surface of the posterior capsule, was not forcibly removed. The anterior hyaloid membrane was not damaged, and no vitreous body was into the AC. Even though a small hole in the anterior hyaloid membrane was observed following surgery, no vitreous incarcerated was observed.

In this case, most of the cortex was found to leak to the posterior surface of the posterior capsule during the operation, and the vitreous body and retina could not be peeped. Therefore, the posterior capsule was torn, and the cortex was sucked out during the operation. A small amount of cortex was closely attached to the posterior surface of the posterior capsule and was not forcibly sucked out, because it would rupture the fragile posterior capsule, with the resultant complications. This operation plan must be gentle to protect the anterior hyaloid membrane, and avoid the rupture of the anterior hyaloid membrane and the vitreous body into the AC, causing the IOP to increase again.

This case report serves as a reminder to clinicians that spontaneous capsule rupture can occur in the anterior and posterior capsules in PLG. When this situation is detected during the operation, the posterior capsule tearing method can be applied to absorb the lens cortex sticking at the posterior surface of the posterior capsule. Noteworthy, a gentle operation is important when tearing the posterior capsule to protect the anterior hyaloid membrane and prevent severe surgical complications.

## Author contributions

**Conceptualization:** Rui-Juan Han, Ai-Jun Tian.

**Data curation:** Yi-Xiang Wu, Mei-Mei Zhou.

**Formal analysis:** Yi-Xiang Wu, Mei-Mei Zhou.

**Investigation:** Rui-Juan Han, Ai-Jun Tian.

**Methodology:** Rui-Juan Han, Ai-Jun Tian.

**Resources:** Yi-Xiang Wu, Mei-Mei Zhou.

**Software:** Ying-Wei Guo, Ming-Yu Ren.

**Writing – original draft:** Ying-Wei Guo.

**Writing – review & editing:** Ming-Yu Ren.
